# Eucaine Lactate

**Published:** 1904-10-15

**Authors:** 


### EUCAINE LACTATE.

The fact that beta-eucaine has failed to supplant cocaine as a local anæsthetic is evidence of the disadvantages attaching to the former; for it is generally recognised that cocaine sometimes gives rise to extremely unpleasant symptoms, and a few cases of fatal syncope attributed to quite small applications of the drug have been reported. It is certain, therefore, that a reliable substitute would meet a distinct want. Professor A. Langgaard,[1] in casting about for a compound which would serve as a good local anæsthetic without sharing the disadvantages attaching to cocaine and beta-eucaine, has found that eucaine lactate appears to fill the necessary requirements. Thus it is soluble in water to the extent of 29 per cent. at ordinary temperatures, it causes neither hyperæmia nor ischæmia, and is quite unirritating, while it acts as an efficient local anæsthetic. If it be desired to produce ischæmia as well as local anæsthesia, this can readily be accomplished by the addition of adrenalin solution. It is to be hoped that a more extended trial of the drug will justify Professor Langgaard's hopes, and we shall look forward with interest to a further report on the subject.

[1] Therapist, Sept. 15, 1904.

